# Regional grey matter volumetric changes in forensic schizophrenia patients: an MRI study comparing the brain structure of patients who have seriously and violently offended with that of patients who have not

**DOI:** 10.1186/1471-244X-8-S1-S6

**Published:** 2008-04-17

**Authors:** Basant K Puri, Serena J Counsell, Nadeem Saeed, Marcelo G Bustos, Ian H Treasaden, Graeme M Bydder

**Affiliations:** 1 MRI Unit, MRC Clinical Sciences Centre, Imaging Sciences Department, Imperial College London, Hammersmith Hospital, Du Cane Road, London W12 0HS, UK; 2 Three Bridges Medium Secure Unit, West London Mental Health NHS Trust, Uxbridge Road, Southall, Middlesex UB1 3EU, UK; 3 Department of Radiology, University of California at San Diego, School of Medicine, 408 Dickinson Street, San Diego, CA 92103-8226, USA

## Abstract

**Background:**

The aim was to carry out the first voxel-based morphometry study of grey matter changes in the whole brain in schizophrenia associated with a history of seriously and violently offending.

**Methods:**

Structural cerebral magnetic resonance imaging scans of 26 patients with schizophrenia were analyzed using voxel-based morphometry: 13 of the patients had seriously and violently offended directly as a result of schizophrenia prior to admission, the offences consisting of homicide, attempted murder or wounding with intent to cause grievous bodily harm; the other 13 patients did not have a history of violence. There was no history of comorbid psychoactive substance misuse disorder in any of the patients. Voxelwise generalized linear modelling was applied to the processed magnetic resonance data using permutation-based non-parametric testing, forming clusters at *t *> 2.3 and testing clusters for significance at *p *< 0.05, corrected for multiple comparisons across space.

**Results:**

The two groups of patients were matched with respect to age, gender and duration of illness, but the group with a history of serious violence was on average receiving a higher dose of antipsychotic medication than the group without a history of violence. There were local regions of reduced grey matter volume in the schizophrenia patient group with a history of serious and violent offending, compared with the schizophrenia patient group without such a history. Significant voxels (*p *< 0.05, corrected for multiple comparisons) were noted bilaterally in the cerebellum and in BA 39 and 40.

**Conclusion:**

These regions are important in verbal working memory. The cerebellum may integrate inputs from ventrolateral prefrontal cortex and parietal regions, providing a corrective signal that refines the process of rehearing the contents of the phonological store. A strong connection has been hypothesized between the supramarginal region corresponding to BA 39/40 and Broca's area, which may correspond largely to the arcuate fasciculus, with the connectional pattern of the language regions of this model fitting the network of parietotemporal-prefrontal connections that participate in working memory. Therefore our results point to the possibility of an abnormality in neural circuits involved in verbal working memory in this group of patients.

## Background

The neurobiology of violence in patients with schizophrenia is becoming an area of increasing academic interest. In their detailed review of neurobiological correlates of violent behaviour in patients with schizophrenia, Naudts and Hodgins [1] point out that evidence has accumulated showing that, compared with the general population, patients with, or who will develop, schizophrenia are at increased risk for violent offending and at even higher risk of committing homicide, with such evidence being derived from investigations of birth and population cohorts comparing the criminality of those with and without schizophrenia, from follow-up studies of schizophrenia patients in the community, from diagnostic studies of representative samples of incarcerated offenders and from investigations of complete cohorts of homicide offenders.

Only a handful of structural neuroimaging studies have been carried out so far to examine whether or not there is a systematic difference in brain grey matter between patients with schizophrenia who have carried out acts of serious violence and patients with schizophrenia who have not done so.

The first published study was that of Chesterman et al. [2], in which 10 male inpatients of a special (high-security) hospital in the south of England underwent cerebral magnetic resonance imaging (MRI). All had committed at least one act of very serious violence, or similarly dangerous acts (e.g. arson). This study reported that the majority of these patients showed reduction in mesial temporal structures. However, not all the patients were diagnosed as suffering from schizophrenia; only six had such a diagnosis, while the remaining four were diagnosed as suffering from a primary personality disorder. Furthermore, there was no direct comparison group.

In the American computed tomography (CT) study by Convit et al. [3], nine male inpatients with schizophrenia with had a history of repetitive violence were compared with nine male inpatients with schizophrenia who had no history of violence. No significant differences were found in cortical atrophy between the two groups, but the Sylvian fissure (lateral sulcus) was subjectively rated as being larger bilaterally in the violent group. No information was available regarding current antipsychotic medication.

In the MRI study of Wong et al. [4], 31 inpatients from a British maximum security hospital were compared with eight normal controls. Of these 31 inpatients, 17 had a history of repetitive violence; they showed a reduction in the volume of the amygdala compared with the normal controls. Similarly, 14 had a history of having committed one violent offence and also showed a reduction in the volume of the amygdala compared with the eight normal controls. Unfortunately the inpatient sample chosen did not have a clear-cut diagnosis of schizophrenia; they were recorded as suffering from either schizophrenia or schizoaffective disorder. Furthermore, it is unclear how many of these inpatients were being treated with antipsychotic medication at the time of the neuroimaging study; presumably the comparison group of eight healthy controls were antipsychotic-naïve.

Barkataki et al. [5] also carried out an MRI study of 13 male inpatients from a British maximum security hospital who had been detained in secure conditions because of their violence (homicide, attempted murder, wounding, robbery, or 'other type of serious violence'). They were compared with a group of 15 male patients with schizophrenia who had no history of serious violence. There was no significant difference in the mean chlorpromazine-equivalent antipsychotic dosage received by each group. Stereological volumetric assessment was conducted using the MEASURE program and Cavalieri method [6], in which a three-dimensional grid of voxel points was overlaid onto a reconstructed three-dimensional MR image that had been reoriented parallel to the anterior-posterior commissure and inter-hemispheric fissure. Using point counting, a rater then manually assessed the volumes of the whole brain, cerebellum, temporal lobe, lateral ventricles, caudate nucleus, putamen, thalamus, hippocampus and amygdala, blind to group. Using this technique, the whole brain was found to have a lower volume and the putamen and amygdala were found to have higher volumes in the violent group; all other comparisons between the two groups were not statistically significant. A further study was carried out on 12 of the 13 inpatients with a history of violence compared with the 15 patient without such a history, to study the cerebral cortex using a cortical pattern-matching method following the manual delineation of 31 sulcal landmarks in each hemisphere of the segmented brain (in which the cerebellum as well as non-brain tissues were removed before analysis) [7]. The violent patients were reported as showing thinning in the right sensorimotor cortex (right M1 and S1) compared with the non-violent patients. A difficulty with these two studies relates to the fact that, in both groups, there was evidence of comorbidity with substance abuse. In particular, in the violent schizophrenia group, two patients had a history of alcohol dependence, one of solvent misuse, two of cannabis dependence, one of alcohol as well as polysubstance misuse (cannabis, ecstasy, LSD and amphetamines) and one of alcohol dependence and polysubstance misuse.

Hoptman et al. [8] manually traced the orbitofrontal cortex on anatomical images from MRI scans carried out at two different American centres as part of a double-blind treatment study comparing the response to four different antipsychotic drugs in chronic antipsychotic treatment-resistant patients. Although imaging parameters and pixel dimensions varied by site, volumes were reported in cubic millimetres, which allowed direct comparisons of data from a total of 49 useable scans across sites. Larger left orbitofrontal cortex grey matter volumes were reported to be associated with greater levels of aggression, recorded using the Overt Aggression Scale, which provides subscales for verbal aggression, physical aggression against objects, physical aggression against self, physical aggression against other people and intervention [9]. Unfortunately, not all the patients had a diagnosis of schizophrenia; some were diagnosed as suffering from schizoaffective disorder, but the results of the scan data from patients with both diagnoses were combined in the publication of the results from this study. Furthermore, over half the sample had a comorbid alcohol use diagnosis (dependence or abuse) and 59% had a comorbid substance use diagnosis.

It can be seen that there have been disadvantages in all these previous studies. These have included, variously: a lack of an appropriate control group; a lack of information about antipsychotic medication, the effect of which on grey matter cannot be ruled out; a lack of a clear-cut single diagnosis of schizophrenia unaccompanied by comorbid psychoactive substance misuse (the effect of which on grey matter cannot be ruled out) or the inclusion of schizoaffective disorder in the group(s) studied; the exclusion of the cerebellum in the analysis; and the use of manual tracing methodologies, which might introduce bias and are likely to have less than a perfect inter-rater reliability. For the present study, therefore, we chose to examine patients with a clear-cut diagnosis of schizophrenia, who did not suffer from any comorbid psychiatric disorder (such as psychoactive substance misuse), and for whom information about antipsychotic medication was available, dichotomized into two groups, one of which had a history of serious violence and the other of which did not. In order to study the grey matter of the brain, including the cerebellum, from MRI scans, without introducing manual tracing methods, we chose to use the unbiased approach afforded by voxel-based morphometry, which requires no *a priori *information about the location of possible differences between groups and which is not operator-dependent. The technique involves spatially normalizing all the images to the same stereotactic space (by registering each of the images to the same template image, by minimizing the residual sum of squared differences between them), segmenting the grey matter from the normalized images, correcting for volume changes arising from spatial normalization and, finally, carrying out a statistical analysis to localize differences between groups; the output from the method is a statistical parametric map which shows regions where grey matter concentration differs significantly between groups [10,11].

We report the first voxel-based morphometry study of grey matter changes in the whole brain in schizophrenia associated with a history of seriously and violently offending.

## Methods

### Subjects

26 patients with a diagnosis of schizophrenia according to DSM-IV underwent cerebral structural MRI. Thirteen of them were inpatients in a medium secure unit. Expert psychiatric opinion, accepted in court, was that all 13 had violently offended directly as a result of schizophrenia prior to admission. These offences consisted of homicide, attempted murder or wounding with intent to cause grievous bodily harm. The other 13 patients were inpatients and outpatients recruited through local London hospitals who did not have a history of violence. There was no history of alcohol dependency in any of the 26 patients and there was no diagnosis of any other comorbid psychoactive substance misuse disorder.

The study was carried out according to the Declaration of Helsinki. The patients were given both verbal and written details of the study and gave written informed consent. The study was approved by the local research ethics committee.

### Image acquisition and processing

High-resolution three-dimensional T_1_-weighted spoiled gradient MR images of the brain of schizophrenia patients with a history of violence were acquired using a 1.5 T Marconi Eclipse system (Marconi Medical Systems, Cleveland, Ohio) at Hammersmith Hospital as a series of 114 contiguous sagittal slices (1.6 mm slice thickness without gaps, 256 × 256 matrix, TR = 30 ms, TE = 3 ms, FA = 30°), and high-resolution three-dimensional T_1_-weighted spoiled gradient MR images of two of the schizophrenia patients without a history of violence were acquired in the same way, while for the remaining patients without a history of violence the high-resolution three-dimensional T_1_-weighted spoiled gradient MR images of the brain were acquired using a 1.0 T Picker HPQ system (Marconi Medical Systems, Cleveland, Ohio) at Hammersmith Hospital as a series of 114 contiguous sagittal slices (1.6 mm slice thickness without gaps, 152 × 256 matrix, TR = 21 ms, TE = 6 ms, FA = 35°). These structural data were analyzed with FSL-VBM, a voxel-based morphometry style analysis [11,12] carried out with FSL tools [13]. First, the values in the T_1_images were scaled to lie between zero and 10 000, and these rescaled structural images were brain-extracted using BET [14]. Next, tissue-type segmentation was carried out using FAST [15]. The resulting grey-matter partial volume images were then aligned to MNI152 standard space using affine registration [16]. The resulting images were averaged to create a study-specific template, to which the native grey matter images were then non-linearly re-registered. The registered partial volume images were then modulated (to correct for local expansion or contraction owing to the non-linear component of the transformation) by dividing by the Jacobian of the warp field. The modulated segmentated images were then smoothed with an isotropic Gaussian kernel with a sigma of 3.5 mm.

### Statistical analyses

Randomized testing with 5000 permutations was used for statistical inference. Voxelwise generalized linear modelling was applied using permutation-based non-parametric testing, forming clusters at *t *> 2.3 and testing clusters for significance at *p *< 0.05, corrected for multiple comparisons across space. Significant clusters were then overlaid on the MNI152 template.

## Results

### Subjects

There was no significant difference between the group with a history of serious violence and the group without a history of violence in respect of mean age (violent group first: 40.4 (standard error 3.7) years; 32.6 (2.5) years), gender (12 men and one woman; 10 men and three women) or mean duration of illness (6.7 (0.4) years; 6.3 (1.3) years), but the average dose of antipsychotic drug used (in chlorpromazine equivalents) was significantly higher in the first group (with a history of serious violence).

### Grey matter

**Figure 1:**
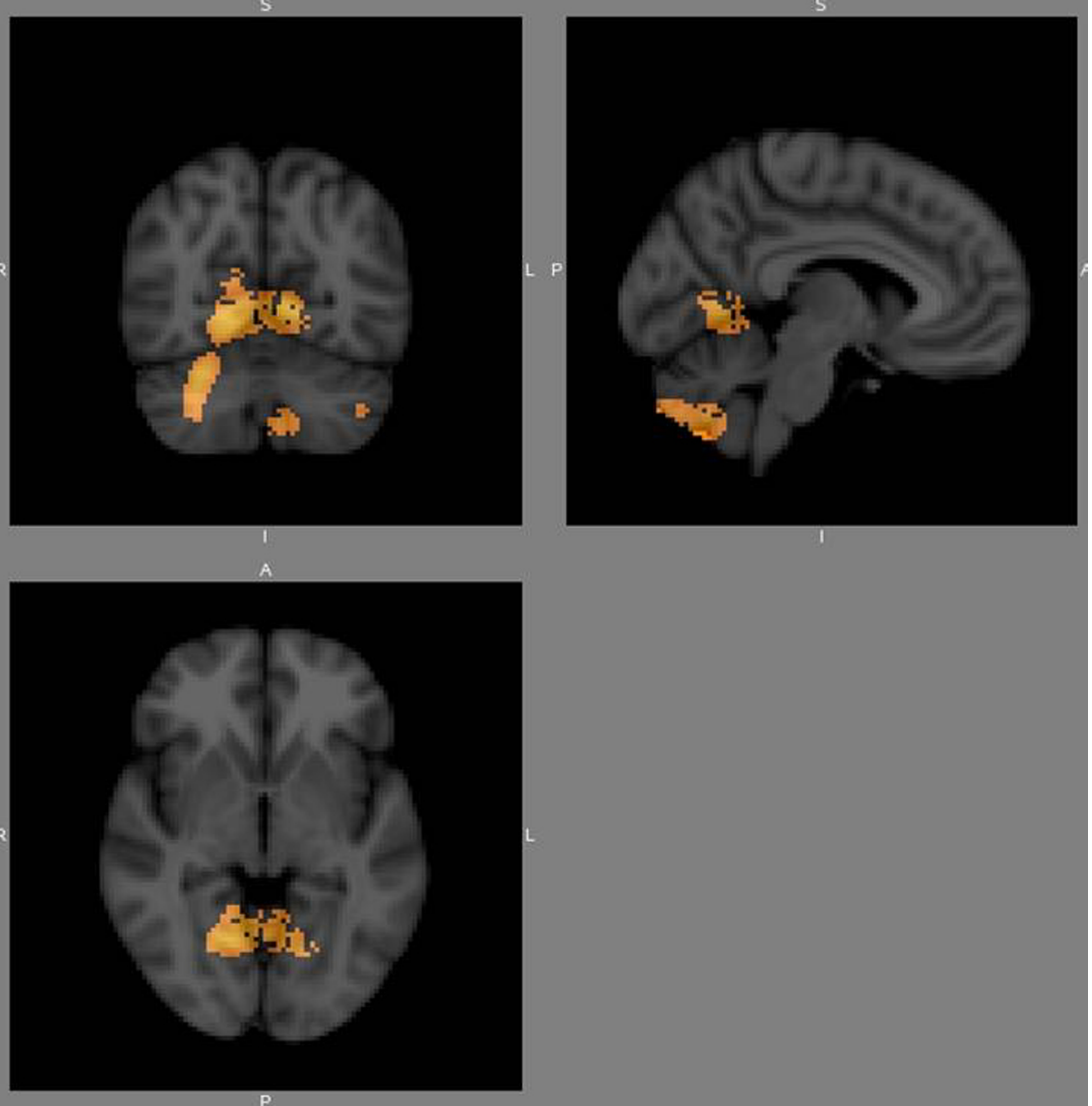
A *p*-value map in which significant clusters, corrected for multiple comparisons, have been overlaid on the MNI152 template.

**Figure 2:**
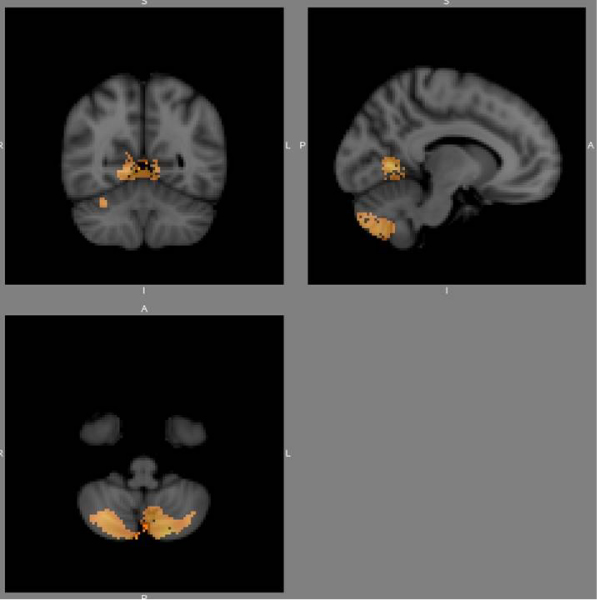
A *p*-value map at a different location from that in Fig. 1, in which significant clusters, corrected for multiple comparisons, have been overlaid on the MNI152 template.

There were local regions of reduced grey matter volume in the schizophrenia patient group with a history of serious and violent offending, compared with the schizophrenia patient group without such a history. Significant voxels (*p *< 0.05, corrected for multiple comparisons) were noted bilaterally in the cerebellum and in BA 39 and 40. Figures 1 and 2 show *p*-value maps in which significant clusters, corrected for multiple comparisons, have been overlaid on the MNI152 template.

## Discussion

This first voxel-based morphometry study of the important group of patients with schizophrenia who are seriously and violently offended has shown two particular bilateral regions of reduced grey matter volume, namely in the cerebellum and in the region around supramarginal gyrus.

While frequently cited early findings, for example in respect of a specific cerebellar involvement in attention, have not been replicated or might be confounded by motor or working memory demands of the respective attention task, there is now convincing evidence for a cerebellar involvement in the mediation of a range of cognitive domains, most notably verbal working memory [17]. In respect of verbal working memory, the cerebellum may integrate inputs from ventrolateral prefrontal cortex and parietal regions, providing a corrective signal that refines the process of rehearing the contents of the phonological store [18,19].

The region around the supramarginal gyrus, in particular BA 40 and BA 39, may also be involved in language function, being an integral part of the network of connectivity for language in the model proposed by Aboitiz and García [20,21], in which there is a particularly strong connection hypothesized between this region and Broca's area; this connection may correspond, at least in large measure, to the arcuate fasciculus [22,23]. The proposed connectional pattern of the language regions of this model fits the network of parietotemporal-prefrontal connections that participate in working memory and indeed Aboitiz and García suggest that language processing is closely related to working memory networks, and that the language regions may have originated from a working memory network for linguistic utterances [20].

Our results therefore suggest that there may be an abnormality in the neural circuits subserving verbal working memory in schizophrenia patients who have seriously and violently offended.

## Conclusion

There is a bilateral reduction in cerebellar and supramarginal gyrus-associated cerebral cortical grey matter in patients with schizophrenia who have seriously and dangerously violently offended. Since these regions are likely to be important in verbal working memory, our results point to the need for further exploration of this cognitive function in this group of patients.

## Competing interests

The authors declare that they have no competing interests.

## Authors' contributions

SJC, GMB and BKP made substantial contributions to the design of the study and were involved in scanning the patients. NS and BKP analyzed the voxel-based morphometry data. IHT and GMB made substantial contributions to the conception of the study. MGB, NS, IHT and BKP were particularly involved in the interpretation of the data. All the authors have been involved in drafting and revising the manuscript and have read and approved the final manuscript.
